# When Important Goals Collide: Prioritization of Prosociality Over Emotion Regulation and Family in Older Adults

**DOI:** 10.1093/geroni/igaf122.345

**Published:** 2025-12-31

**Authors:** Nicole Long-ki Fung, Molly Lin, Hongmei Lin, Helene Fung

**Affiliations:** The Chinese University of Hong Kong, Hong Kong, China (People’s Republic); University of Zurich, Zürich, Switzerland; The Chinese University of Hong Kong, Hong Kong, China (People’s Republic); The Chinese University of Hong Kong, Hong Kong, China (People’s Republic)

## Abstract

The socioemotional selectivity theory (Carstensen et al., 1999) suggests that as future time perspective becomes more limited in old age, motivational priorities shift toward emotionally meaningful goals. For instance, compared with younger adults, older adults become more prosocial (Bailey et al., 2020), prefer their own family members (Fung et al., 1999), and put higher emphasis on emotional regulation (Isaacowitz et al., 2021). However, these important goals are not always compatible with one and other. It remains unclear how older adults will prioritize these important goals when they are in conflict. To address this question, we recruited with 144 older (M = 67.8, SD = 5.20, 60.4% female) and 130 younger adults (M = 24.0, SD = 4.44, 74.6% female) for two studies. Participants read hypothetical scenarios where important goals compete. In Study 1, participants decided whether they would engage in prosocial behaviors with less emotional value over pleasant activities with less prosocial value (prosocial vs. emotional regulation). Older adults prioritized being prosocial over emotional regulation (t = 4.02, p < .001), while such tendency was not observed in younger adults. In Study 2, participants decided whether they would choose to help others at the expense of their family over benefiting their family against the welfare of others (prosocial vs. profamily). Again, older adults prioritize being prosocial over benefiting family (t = 9.14, p < .001), while younger adults did not show such a preference. The findings suggest that prosocial goal may take precedence in the motivational shift in late adulthood.

